# A cross-sectional study exploring the predictors of herpes zoster vaccination for people aged over 50 years old in Chaoyang district, Beijing

**DOI:** 10.3389/fpubh.2024.1486603

**Published:** 2025-01-23

**Authors:** Jiao Zhang, Shuo Zhang, Bin Jia, Yunhua Bai, Zhen Li, Fang Liu, Yingxue Hu, Xiaojing Guo, Jianxin Ma, Shuming Li, Qian Shi

**Affiliations:** ^1^Beijing Chaoyang District Center for Disease Control and Prevention, Beijing, China; ^2^State Key Laboratory of Molecular Vaccinology and Molecular Diagnostics, School of Public Health, Xiamen University, Xiamen, Fujian, China; ^3^Institute of Basic Medical School, Chinese Academy of Medical Science and Peking Union Medical College, Beijing, China

**Keywords:** herpes zoster, the herpes zoster vaccine, vaccination coverage, predictor, questionnaire survey

## Abstract

**Background:**

Vaccination is an effective measure to prevent herpes zoster and its related complications. The coverage of herpes zoster vaccination is extremely low in China, and there is a notable lack of research investigating the barriers to promoting the herpes zoster vaccine in China.

**Objectives:**

This study aims to survey the status of herpes zoster (HZ) vaccination and its associated factors among individuals aged 50 years and older, it also seeks to identify the barriers to vaccination and dissemination, thereby providing a scientific foundation for enhancing the vaccination rate of herpes zoster.

**Methods:**

From March to August 2023, a questionnaire survey was conducted using a multi-stage sampling method on permanent residents aged 50 years and older in Chaoyang district of Beijing. Logistic regression analysis and decision tree models were employed to explore the predictors herpes zoster vaccination behavior.

**Results:**

The herpes zoster vaccination coverage was 13.26% (403/3040), with 52.4% of respondents expressing willingness but not yet receiving the vaccine, while 34.31% (1,043/3040) were unwilling to be vaccinated. Among those willing but not vaccinated, 48.06% cited high cost as the reason for abstaining from vaccination. Multivariate logistic regression analysis revealed that certain factors were associated with lower vaccination coverage, including being female, having a low frequency of influenza episodes (less than twice per year), and having chronic diseases. On the other hand, higher vaccination coverage was observed among individuals whose spouses had a master’s degree or higher, those awarded that a history of chickenpox may lead to potential herpes zoster infection, those who were knowledgeable about the herpes zoster vaccine, and those received recommendations from medical staff. Additionally, the decision tree model confirmed that recommendations from medical staff had the most significant impact on vaccination.

**Conclusion:**

The survey reveals a generally low vaccination coverage of the herpes zoster vaccine among residents aged over 50 in this area. It is recommended to enhance the role of medical staff in advocating for vaccination, conduct community-based educational initiatives that focus on varicella, herpes zoster, and related knowledge, and alleviate the financial burden to improve the herpes zoster vaccination coverage.

## 1 Introduction

Herpes zoster (HZ) is an infectious dermatological condition caused by the reactivation of Varicella-zoster virus (VZV), which remains latent in the posterior root ganglia or cranial ganglia of the spinal cord following primary infection (1). The primary clinical manifestations encompass clusters of vesicles distributed unilaterally along the dermatomal strip, accompanied by pain. In severe instances, it can also give rise to postherpetic neuralgia and lead to complications such as visual impairment, blindness, auditory abnormalities, and vertigo, significantly impacting the quality of life among older adult patients (2). Post-herpetic neuralgia (PHN) is the most common complication of herpes zoster. The pain can last for months, or even longer in severe patients, and seriously affects the quality of life of patients (3). Herpes zoster not only affects the physical and mental health of patients, resulting in decreased quality of life but also brings serious disease burden and public health concern to families and society.

The incidence of herpes zoster in China has exerted significant strain on the allocation of medical resources, as evidenced by the surge in patient numbers, hospital visits, hospitalization rates, and recurrence rates. Based on research findings, the global prevalence of herpes zoster is estimated to be approximately 3–5 cases per 1,000 individuals annually (4), while in the Asia-Pacific region, it ranges from 3 to 10 cases per 1,000 people (5). According to a systematic review, the annual incidence of herpes zoster in China is reported as 4.28/1,000 in the general population and 11.69/1,000 among individuals aged over 60 years (6). It is estimated that a minimum of 2.77 million cases of herpes zoster occur annually in China, with approximately 92.2% necessitating medical intervention. It is estimated that more than 55,000 people develop herpes zoster each year in Beijing alone (7). Furthermore, older patients may have more severe symptoms (8). The incidence of herpes zoster is progressively increasing each year due to the demographic shift towards an aging population. The hospitalization rate of herpes zoster patients worldwide ranges from 2 to 25 per 100,000 individuals, whereas in China, the corresponding rate is 6 per 100,000 individuals (6). The average duration of hospitalization for the initial episode of herpes zoster was 21.6 days (9).

The prevalence of herpes zoster among the older adult has emerged as a significant public health concern. Vaccination against herpes zoster represents a crucial preventive measure, exhibiting commendable immunogenicity and economic advantages. Consequently, several developed nations have incorporated adult herpes zoster vaccination programs into their healthcare systems. Notably, the overall efficacy of administering two doses of the herpes zoster vaccine was found to be 85.5% (95%CI: 83.5–87.3%) (10). Even among immunocompromised individuals, the herpes zoster vaccine can still reduce the risk of developing the disease (11). Consequently, enhancing the vaccination coverage rate against herpes zoster can effectively mitigate the occurrence of herpes zoster (12). The safety and efficacy of the vaccine have been fully demonstrated, however, significant disparities in vaccination rates persist (13).

The coverage of herpes zoster vaccination varies greatly among different populations. In 2023, our team conducted a survey among patients with chronic diseases in Chaoyang district and showed that the vaccination coverage rate was 9.95% (14). The literature shows that, the herpes zoster vaccination coverage rates among individuals aged 60 years and above in Beijing, China, from 2020 to 2022 were recorded as 0.11, 0.25, and 0.54% (15), Respectively, while the corresponding rate for this age group in the United States was reported as 41.1% in 2021 (16). In the UK, during the year 2022, herpes zoster vaccination coverage rates ranged from 44% to a high of 80.7% among individuals aged between70 and 80 years old. The variations may be ascribed to regional disparities in healthcare systems, vaccination policies, accessibility, and implementation efforts, alongside public acceptance of vaccine pricing and hesitancy towards the vaccine itself, encompassing doubts regarding its efficacy and concerns about potential adverse effects. Currently, the phenomenon of vaccine hesitancy has endangered the vaccination of people, seriously affecting human health. In 2019, the World Health Organization listed vaccine hesitancy as one of the top ten threats to global health (17). A systematic review of vaccination willingness across various regions worldwide indicates that only half of the population is willing to receive the shingles vaccine. This suggests that there is still a significant lack of public knowledge regarding vaccines (18). Among the unvaccinated individuals with chronic conditions in this region, 56.75% expressed a willingness to receive vaccination (14). Investigating the influencing factors pertaining to herpes zoster vaccination behavior is crucial for gaining deeper insights into population immunization patterns and providing a scientific basis for developing targeted public awareness campaigns and educational programs aimed at improving vaccination rates while reducing both the incidence and prevalence of herpes zoster infection—ultimately contributing towards ensuring public health (19, 20), and leading to economic benefits (21).

It is well known that vaccination knowledge, attitude and education level of vaccination recipients affect vaccination behavior. In order to further explore what factors affect vaccination behavior, the authors put forward a meaningful hypothesis to explore the potential associations between herpes zoster vaccination behavior and demographic characteristics, education level, chronic disease status, and receiving vaccination advice from health care providers.

Despite the existence of several studies on herpes zoster vaccine questionnaires (22, 23), prior research has primarily concentrated on demographic characteristics and disease awareness. Investigations into the proactive role of healthcare providers in promoting vaccination remain scarce. It is essential to examine the public’s knowledge gaps regarding the disease and vaccination to inform the development of more targeted health education initiatives and to provide a foundation for the government to formulate integrated healthcare and prevention strategies.

## 2 Materials and methods

### 2.1 Research design and respondents

An online questionnaire survey was conducted from March 16 to July 23, 2023. Residents aged 50 years and above were invited to participate in the survey. Inclusion criteria: (1) residents aged ≥50 years in Chaoyang district of Beijing; (2) Being able to communicate normally without obvious cognitive impairment; and (3) Informed consent was obtained from the respondents. According to the sample size formula necessary for the cross-sectional survey: $N={\left(\frac{z_{1-\frac{\alpha }{2}}\times \sqrt{p\times \left(1-p\right)}}{d}\right)}^{2}$ . Based on the previous survey conducted in Beijing, the vaccination rate was approximately 0.54%, the significance level *α* was 0.05, the allowable error d was 0.50p, and the sample size was approximately 2,830 individuals. Considering the potential non-response rate, we distributed a total of 3,500 questionnaires.

The electronic questionnaire was distributed and recovered through the network platform named Questionnaire Star.^1^ The respondents filled in the questionnaire by scanning the two-dimensional code of the questionnaire with their mobile phones. This investigation is in accordance with the 1975 Declaration of Helsinki.

### 2.2 Questionnaire survey

The questionnaire was written in Chinese, the content was comprehensive of the influencing factors in previous literature, and was reviewed by experts. Then we distributed questionnaires to 20 people from the communities for pre-survey,10 were from urban areas and 10 were from urban–rural junctions. After the pre-survey, we collected the problems existing in the questionnaire through interviews, such as some options were not well understood by residents so we added notes after the options and then distributed the questionnaire.

The content of the questionnaire mainly included five parts. The first part is the sociological characteristics of the respondents, such as age, gender, education, and occupation, living in urban or urban–rural junction areas; The second part was family factors such as marital status, spouse’s education background, and the number of cohabitants; The third component included health-related factors, such as frequency of influenza, chronic diseases, history of herpes zoster, history of varicella, herpes zoster prevalence in a household member or person with whom they knew well, and whether a medical stuff recommended herpes zoster vaccination; The fourth part was the cognition about whether they had heard of herpes zoster, whether they knew that people with varicella could get herpes zoster, whether they had heard of herpes zoster vaccine, and how to get the knowledge of the herpes zoster vaccine; The fifth part was the vaccination status/willingness, as well as factors such as service accessibility and economic accessibility (monthly income, acceptance of vaccination price). After the reliability and validity test, the questionnaire showed excellent reliability (Cronbach’s *α* = 0.926) and construct validity (KMO = 0.966, Bartlett’s sphericity tests *p* < 0.001).

A multi-stage sampling method was employed in this study. Initially, the random number table method was employed to select 8 urban areas and 6 urban–rural junction areas from a total of 43 areas (24 urban areas and 19 urban–rural junction areas) within the Chaoyang district of Beijing.

Before distributing the questionnaire, the staff underwent training to ensure that respondents comprehended the purpose and significance of the study. It was then left to the discretion of respondents whether or not they wished to proceed with completing the questionnaire. Additionally, we implemented measures in our questionnaire where each respondent’s IP address was recorded to prevent duplicate submissions. Furthermore, verbal informed consent was obtained from participants, stating that their participation would remain anonymous to minimize information bias. The entire process of completing the questionnaire typically took approximately ten minutes.

### 2.3 Statistical methods

The data were exported from the Questionnaire Star platform after collecting the questionnaires. Data analysis and visualization are completed by SPSS20.0 and R (version 4.3.3). For count data, frequency and percentage were calculated, and the chi-square test was employed for univariate analysis. A multivariate logistic regression model using a stepwise selection method was used to analyze (α_in_ =0.05, α_out_ =0.10). Additionally, we utilized the conditional Inference Decision Tree algorithm classification model to perform the analysis.

## 3 Results

### 3.1 Demographic characteristics of respondents

A total of 3,500 people were surveyed, with 3,040 valid questionnaires returned and a response rate of 86.86%. The assignment of all the demographic characters was presented in Table 1. Among the respondents, 1,366 (44.9%) were recruited from urban areas and 1,674 (55.1%) from urban–rural junctions. The male participants accounted for 1,136 (37.37%), while the female participants constituted 1904 (62.63%). Regarding age distribution, the majority of participants fell within the range of 50–59 years old, comprising 1,618 individuals (53.22%). Additionally, there were 940 individuals (30.92%) aged between 60 and 69 years, followed by 397 individuals (13.06%) aged between 70 and 79 years, and finally, a smaller group of older adult participants ≥80 years old consisting of only 85 individuals (2.80%). The median age was calculated as 66 years, with an age range spanning from 50 to 91 years. In terms of educational background, most of the respondents had obtained a bachelor’s degree or below; specifically, this category encompassed 2,962 people, which accounted for about 97.43% of the total sample size.

**Table 1:** The independent variable assignment in influencing factors exploring.

Independent	Assignment
Community	1 = urban; 2 = urban–rural junctions
Gender	1 = Male; 2 = Female
Marital status	1 = Unmarried, Divorced, Widowed or Others; 2 = Married
Age	1 = 50 ~ 59; 2 = 60 ~ 69; 3 = 70 ~ 89
Educational background	1 = Bachelor’s degree and below; 2 = Master’s degree and above
Job	1 = Medical staff; 2 = Others
Spouse’s educational background	1 = Bachelor’s degree and below; 2 = Master’s degree and above
Presence of chronic illness	0 = No; 1 = Yes
Frequency of flu infections	1 = Never; 2 = Hardly (Once every several years); 3 = Sometimes (Once a year); 4 = Often (At least twice a year)
Monthly Income	1 = <10,000; 2 = ≥10,000
Knowing that having had chickenpox may lead to herpes zoster	0 = No; 1 = Yes
Number of co-residents	1 = 1 ~ 2; 2 = ≥3
Heard of the herpes zoster vaccine	0 = No; 1 = Yes
Recommendation from medical staff for vaccination	0 = No; 1 = Yes
Having had chickenpox	0 = No; 1 = Yes
Having had herpes zoster	0 = No; 1 = Yes
Co-residents having had chickenpox	0 = No; 1 = Yes

According to the survey results, a total of 619 individuals (20.4% of the sample) reported having experienced varicella, and 458 individuals (15.07%) had a history of herpes zoster. 792 individuals (26.05%) had cohabitants or close acquaintances with a documented history of herpes zoster. Furthermore, among these respondents, 1,674 individuals (55.07%) rarely or never suffered from influenza, while 1,366 individuals (44.93%) experienced influenza more than once per year. Additionally, chronic diseases were reported by 1,178 respondents (38.75%), with hypertension being the most common condition affecting 795 individuals (26.15%), followed by hyperlipidemia in 515 individuals (16.94%), and diabetes in 368 individuals (12.11%).

### 3.2 Awareness rate of herpes zoster and herpes zoster vaccine

2,463 (81.02%) respondents reported awareness of herpes zoster, while 792 (26.05%) respondents were knowledgeable about the association between varicella and herpes zoster. Additionally, 1,652 (54.34%) participants had known the herpes zoster vaccine, and 1,203 (39.57%) individuals had received recommendations from medical staff. Among the unvaccinated respondents, 59.92% (1,580/2637) expressed their dissatisfaction with the current price of Recombinant Zoster Vaccine (CHO cell) at 1598 RMB per dose for a total of two doses. Most of the respondents, accounting for 79.77% (2,425/3040), expressed their desire for a vaccine price lower than 300 RMB per dose.

**Figure 1:**
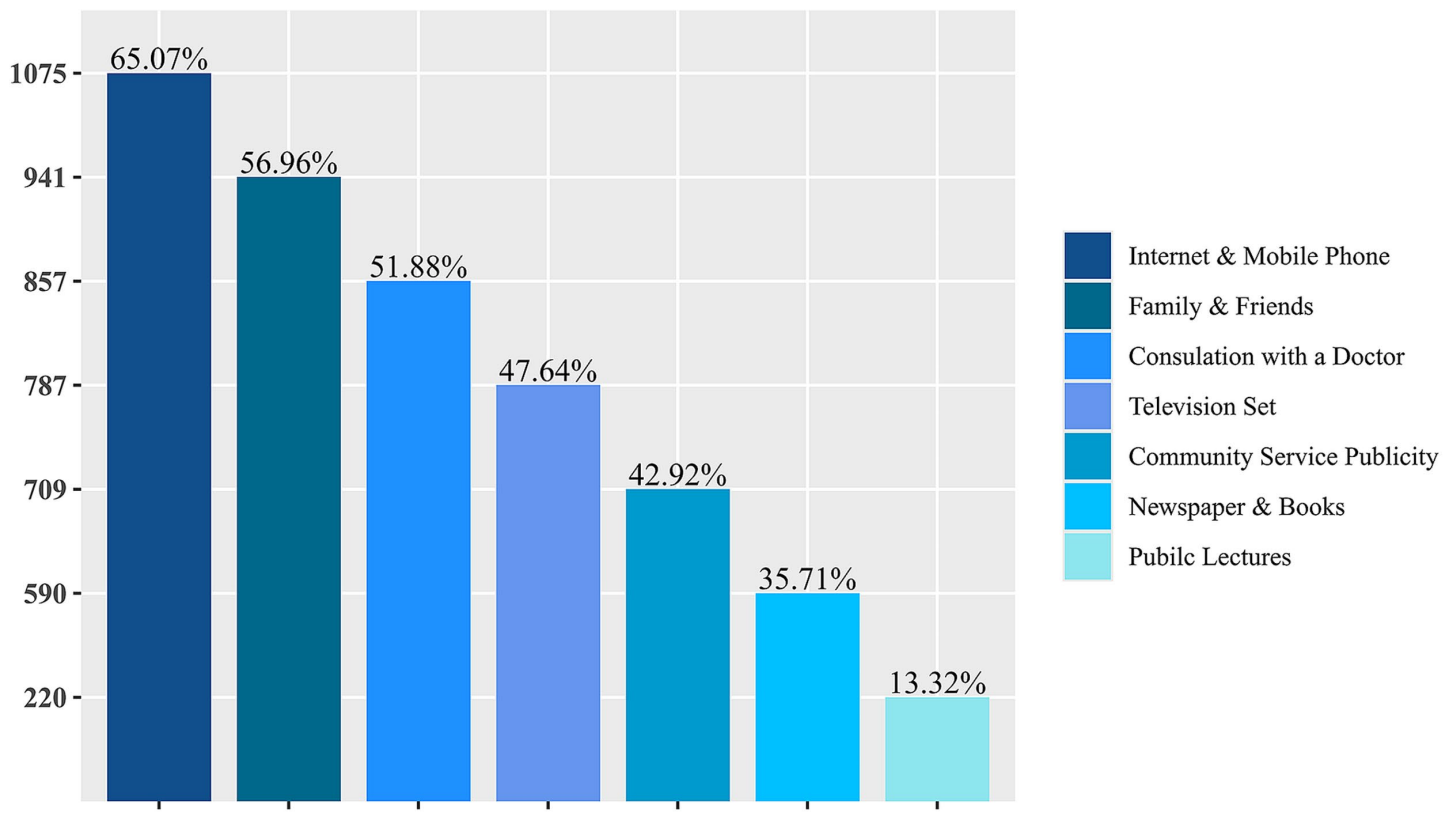
Access to information regarding the herpes zoster vaccine.

The findings presented in Figure 1 indicated that the primary sources of information about the vaccine were the internet and mobile phone (65.07%), family and friends (56.96%), doctor consultations (51.88%), television (47.64%), and community public service publicity (42.92%) (see Figure 1).

### 3.3 The subjects’ willingness to receive the herpes zoster vaccine

Among the participants, 403 individuals (13.26%) had received herpes zoster vaccination, while 1,594 individuals (52.43%) expressed willingness to be vaccinated but had not yet received it, and 1,043 individuals (34.31%) were unwilling to be vaccinated (see Table 2).

**Table 2:** Willingness to receive herpes zoster vaccination.

Willingness	Frequency (*n*)	Percentage (%)
Willingness to receive herpes zoster vaccination		
Willing to and already vaccinated	403	13.26
Willing but not yet vaccinated	1,594	52.43
Not willing	1,043	34.31
Reasons for willingness to receive herpes zoster vaccination		
Enhance resistance and prevent infectious diseases	1745	87.38
Reduce expenses due to illness	947	47.42
Recommendations from families or friends	481	24.09
Government suggestion	496	24.84
Recommendation from medical staff	448	22.43
Reasons for not receiving the herpes zoster vaccine yet		
Too expensive	766	48.06
Lack of information	701	43.98
Logistical challenges associated with vaccination (such as transportation and scheduling constraints)	526	33.00
Reasons for unwillingness to receive the herpes zoster vaccine		
Believing that physical health does not require vaccination	415	39.79
Worrying about side effects	344	32.98
Too expensive	263	25.26
Acceptance of price		
Totally unacceptable	998	33.46
Unacceptable	582	6.41
Neutral	724	17.82
Relatively acceptable	175	6.41
Very acceptable	158	6.67
Acceptable price range		
<300￥/dose	2,425	79.77
300 ~ 500￥/dose	455	14.97
500 ~ 1,000￥/dose	112	3.68

### 3.4 Univariate analysis of herpes zoster vaccination behavior

The coverage of herpes zoster vaccine was 13.26% (403/3040). Univariate analysis revealed significant variations among variables including gender, age, education, occupation, marital status, spouse’s education level, monthly income, frequency of influenza experienced by individuals, awareness of the association between varicella and herpes zoster, and their knowledge about the herpes zoster vaccine. Moreover, the history of chronic diseases and recommendations from medical staff were also found to influence vaccination behavior (see Table 3).

**Table 3:** Vaccination coverage and univariate analysis of herpes zoster vaccine among residents aged 50 and above in Chaoyang district, Beijing.

Variable	Investigated	Vaccinated	Vaccination coverage	Univariate analysis	
				χ^2^	*p*
Gender					
Male	1,136	188	16.55	17.101	<0.001
Female	1904	215	10.85		
Age					
50–59 years old	1,618	257	15.88	21.585	<0.001
60–69 years old	940	102	10.85		
70 years old	482	44	9.13		
Educational background					
Bachelor’s degree and below	2,962	381	12.86	15.557	<0.001
Master’s degree or above	78	22	28.21		
Job					
Medical staff	321	65	20.25	15.261	<0.001
Others	2,719	338	12.43		
Marital status					
Unmarried, divorced, widowed or others	340	58	17.06	4.813	0.028
Married	2,700	345	12.78		
Spouse’s educational background					
Bachelor’s degree and below	2,762	336	12.17	10.126	0.001
Master’s degree or above	95	22	23.16		
Monthly household income					
≤10,000	2,763	341	12.34	22.074	<0.001
>10,000	277	62	22.38		
Frequency of flu infections					
Often (at least twice a year)	213	51	23.94	23.886	<0.001
Sometimes (once a year)	1,153	148	12.84		
Hardly (once every several years)	1,308	154	11.77		
Never	366	50	13.66		
Knowing that having had chickenpox may lead to herpes zoster.					
Yes	792	168	21.21	58.949	<0.001
No	2,248	235	10.45		
Heard of the herpes zoster vaccine					
Yes	1,652	296	17.92	68.360	<0.001
No	1,388	107	7.71		
Presence of chronic illness					
Yes	1,178	117	9.93	18.485	<0.001
No	1862	286	15.36		
Recommendation from medical staff for vaccination					
Yes	1,203	269	22.36	143.497	<0.001
No	1837	134	7.29		
Total	3,040	403	13.3		

### 3.5 Multivariate logistic regression analysis of herpes zoster vaccination behavior

All variables collected in the survey were included as independent variables in the multivariate logistic regression analysis, with the absence of multicollinearity (Variance Inflation Factor, VIF < 5). Herpes zoster vaccination (0 = no, 1 = yes) was the dependent variable, and the independent variables were listed in Table 1. The model fitting was performed using the backward LR method (with an inclusion criterion of *α* = 0.05 and an exclusion criterion of *β* = 0.1). The Hosmer-Lemeshow test indicates the *p*-value was 0.396 and the 2-Log likelihood value was 1546.099, which meant that the model fits well.

The results of the multivariate logistic regression analysis indicated that females had a lower vaccination coverage for the herpes zoster vaccine (OR = 0.584, 95% CI: 0.447–0.762). Those who did not frequently suffer from flu are also less likely to receive the vaccine (e.g., for those who occasionally get influenza—once a year—OR = 0.325, 95% CI: 0.204–0.517). Furthermore, individuals with chronic diseases demonstrated a lower vaccination coverage (OR = 0.653, 95% CI: 0.491–0.868).

**Figure 2:**
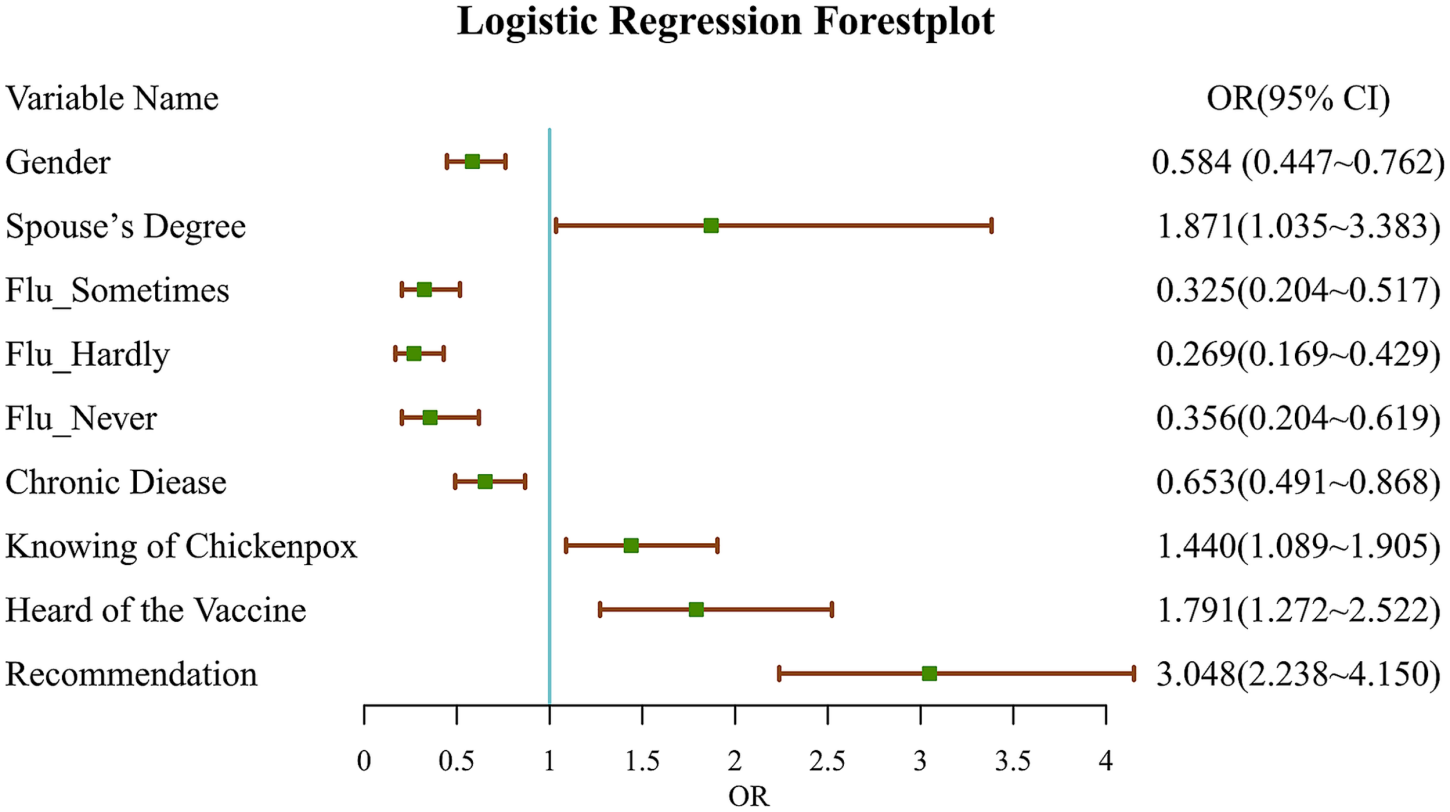
Logistic regression forest plot, focuses on OR(95%CI). Gender: based on male; spouse’s degree: based on bachelor or below; Flu_Sometimes: catching flu once a year, based on catching flu more than twice a year; Flu_Hardly: catching flu once every several years, based on catching flu more than twice a year; Flu_never: never get flu, based catching flu more than twice a year; chronic disease: presence of chronic illness, based on no; knowing of chickenpox: knowing that having had chickenpox may lead to herpes zoster, based on no; heard of the vaccine: based on no; recommendation: recommendation from medical staff for vaccination, based on no.

Conversely, individuals whose spouse had a master’s degree or higher are more likely to be vaccinated (OR = 1.871, 95% CI: 1.035–3.383). Those who were aware that a person who has had chickenpox may develop herpes zoster also have a higher vaccination coverage (OR = 1.440, 95% CI: 1.089–1.905). Additionally, individuals who have heard of the herpes zoster vaccine are more likely to be vaccinated (OR = 1.791, 95% CI: 1.272–2.522), and those who have received a recommendation from the medical staff are significantly more likely to be vaccinated (OR = 3.048, 95% CI: 2.238–4.150) (see Table 4; Figure 2 for details).

**Table 4:** Unconditional logistic regression analysis of factors influencing herpes zoster vaccine uptake behavior among survey respondents.

	B	SE	Waldχ^2^	*p* value	OR(95%CI)	VIF
Gender (with males as the reference group)	−0.538	0.136	15.178	<0.001*	0.584(0.447, 0.762)	1.022
Spouse’s educational background (with a bachelor’s degree or lower as the reference group)	0.627	0.302	4.298	0.038	1.871 (1.035, 3.383)	1.014
Frequency of flu infections (With two times or above as the reference group)			30.874	<0.001*		1.013
Sometimes (Once a year)	−1.124	0.237	22.424	<0.001*	0.325 (0.204, 0.517)	
Hardly (once every several years)	−1.314	0.238	30.705	<0.001*	0.269 (0.169, 0.429)	
Never	−1.034	0.283	13.351	<0.001*	0.356 (0.204, 0.619)	
Presence of chronic illness (with no as the reference group)	−0.426	0.145	8.619	0.003	0.653 (0.491, 0.868)	1.031
Knowing that having had chickenpox may lead to herpes zoster (with no as the reference group)	0.365	0.143	6.525	0.011	1.440 (1.089, 1.905)	1.152
Heard of the herpes zoster vaccine (with no as the reference group)	0.583	0.175	11.153	0.001	1.791 (1.272, 2.522)	1.289
Recommendation from medical staff for vaccination (with no as the reference group)	1.114	0.158	50.033	<0.001*	3.048 (2.238, 4.150)	1.277
Intercept	−1.140	0.333	11.715	0.001	0.320	

### 3.6 Analyzing the influencing factors of herpes zoster vaccination behavior by decision tree model

A decision tree model was constructed using the conditional inference decision tree algorithm, with the vaccination of herpes zoster vaccine (vaccination = 1, no vaccination = 0) as the dependent variable. A total of 11 nodes and 6 end nodes were established in the model while screening out five explanatory variables including medical staff recommendation for herpes zoster vaccine vaccination, influenza status, marital status, whether knowing individuals who had chickenpox can get herpes zoster, and gender.

The variable in the initial layer was whether herpes zoster vaccination was recommended by a medical staff, indicating that it had the strongest effect with herpes zoster vaccination. Individuals who received advice from medical staff had significantly higher vaccination coverage compared to those who did not, with rates of 22.4 and 7.3%, respectively.

Among individuals who received advice from their healthcare providers, marital status emerged as a significant influencing factor. The vaccination coverage was higher among unmarried and divorced individuals (44.3%) compared to married and other status individuals (21.2%). Within the group of Married and other status individuals who were advised by their medical staff to get vaccinated, gender played a role in determining the vaccination coverage, with males (27.4%) exhibiting a higher rate than females (17.4%).

**Figure 3:**
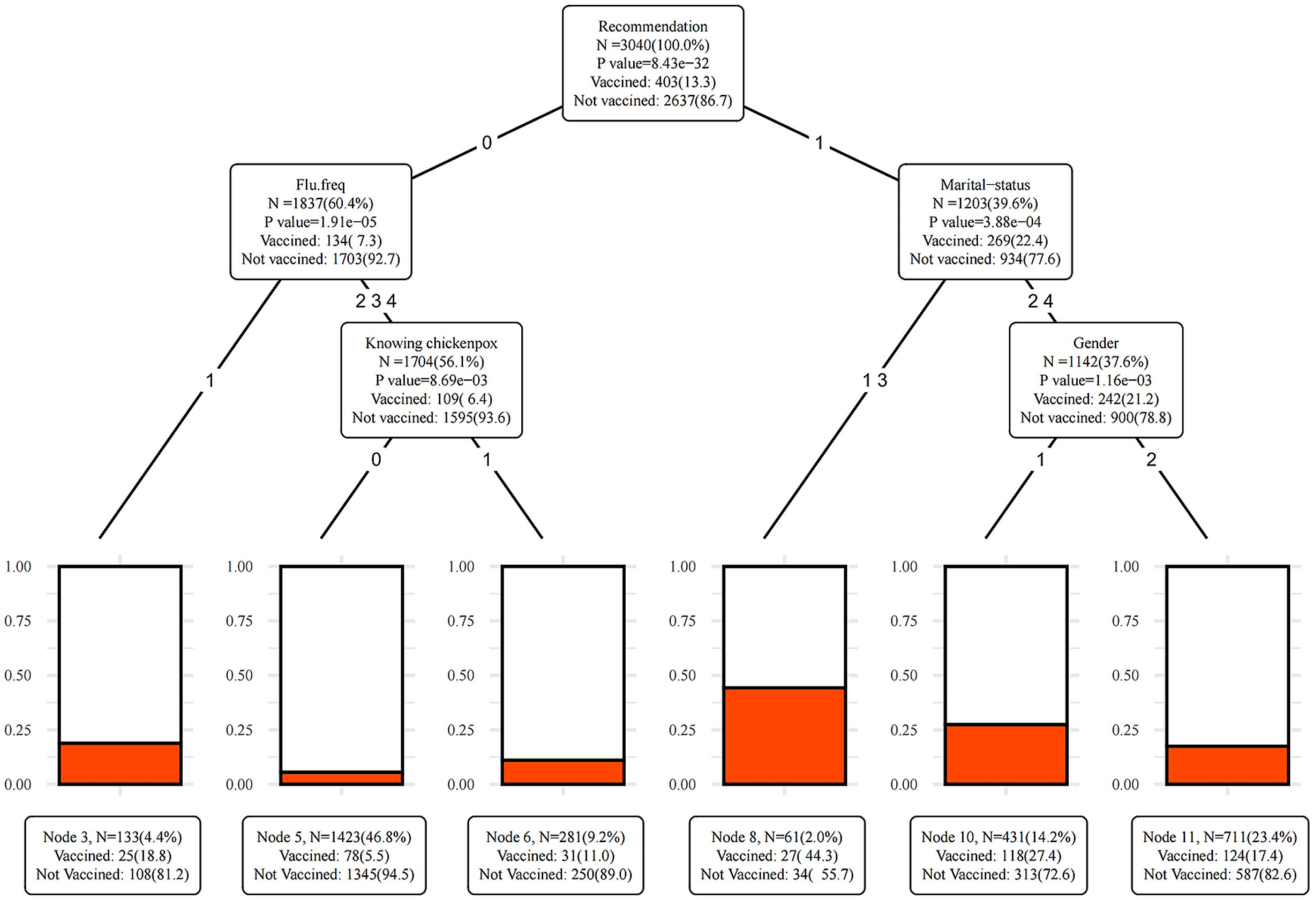
The decision tree model result. Recommendation: recommendation from medical staff for vaccination; flu.freq: frequency of flu infections; knowing chickenpox: knowing that having had chickenpox may lead to herpes zoster. In Recommendation, 0 means No, 1 means Yes. In Flu.freq, 1 means Often, 2,3,4 means Sometimes, Hardly, and Never. In Knowing chickenpox, 0 means No, 1 means Yes. In marital-status, 1 and 3 means unmarried and divorced, 2 and 4 means married and widowed or others. In gender, 1 means male, 2 means female.

Among individuals not recommended for vaccination, the frequency of influenza was significantly associated with the vaccination coverage. Those experiencing influenza twice or more per year had a higher vaccination coverage (18.8%) compared to those with influenza once or less per year (6.4%). Among individuals without medical advice and infrequent occurrences of influenza (less than once a year), those aware of the association between varicella and herpes zoster were more likely to receive the vaccination (11.0%) than those unaware (5.5%) (see Figure 3).

## 4 Discussion

This study conducted a cross-sectional survey of residents aged above 50 in Chaoyang district, Beijing. The results showed that the vaccination coverage for herpes zoster vaccine was 13.26%, which was higher than the vaccination coverage in other regions of China (24), but much lower than the results of the coverage of Australia and England, (25, 26). 52.43% of people are willing but have not yet received the herpes zoster vaccine, which is higher than 43.79% in Shanghai, China (27), and 42.07% in Zhengzhou, China (28). Exploring the influencing factors of herpes zoster vaccination behavior among people aged above 50 in Chaoyang district is of great significance for improving vaccination coverage in the area. In the statistical analysis method, we combined the decision tree model and the logistic regression model. Logistic regression excels in analyzing linear relationships, whereas decision trees are adept at handling nonlinear relationships. Logistic regression surpasses decision trees in capturing the overall structure of data, while decision trees outperform logistic regression in analyzing local data patterns. Therefore, we used two methods to analyze the factors that affect the vaccination behavior of the herpes zoster vaccine, to provide more accurate control measures. After conducting an analysis, we discovered that factors such as whether healthcare workers recommended the herpes zoster vaccine, frequency of influenza, marital status, whether individuals were aware that those who had chickenpox could be infected with herpes zoster, and gender might influence the vaccination behavior for the herpes zoster vaccine.

In this study, the recommendations of medical staff have a significant impact on vaccination behavior. The result of the decision tree model indicates that the recommendation of vaccination by medical staff had the greatest impact on vaccination behavior. The logistic regression analysis revealed that the vaccination rate among individuals with vaccine recommendation was threefold higher compared to those without vaccine recommendation. The results align with those of a recent systematic review examining COVID-19 vaccination in children with the findings that recommendations from healthcare professionals were a key facilitator of vaccination. This indicates trust in healthcare providers is generally high, and therefore their recommendations are powerful, especially in all areas of vaccination (29). A key important point is that healthcare providers essentially provide cues to act. So, we can leverage professional healthcare providers to recommend vaccines to improve vaccination uptake (30), which is the simplest and most effective method.

Logistic regression analysis and decision tree modeling revealed that individuals who experienced influenza two or more times per year exhibited a higher likelihood of receiving influenza vaccination. Previous studies have demonstrated that individuals with a greater understanding of influenza and its vaccine are more inclined to seek immunization against the virus (31, 32). However, no study has explored the relationship between influenza frequency and the utilization of vaccines other than those specifically targeting influenza. Therefore, we can infer that individuals with a higher incidence of influenza may proactively educate themselves on preventive measures against infectious diseases such as influenza. Conversely, they may also visit healthcare facilities more frequently for treatment purposes, leading to increased interactions with medical professionals and enhanced opportunities for acquiring knowledge related to disease prevention. Consequently, their awareness regarding both influenza and other infectious diseases as well as preventive strategies is likely to be heightened. Nevertheless, further investigation is required to elucidate the underlying reasons behind this finding. In contrast, individuals experiencing lower incidences of influenza may develop complacency towards their health status and underestimate their susceptibility to infectious diseases.

People with a higher level of knowledge regarding varicella and zoster diseases and vaccines exhibited elevated vaccination rates. The findings revealed that individuals aged 50 years and above who were aware of the potential link between prior varicella infection and herpes zoster demonstrated higher vaccination rates compared to those lacking awareness, with respective rates of 21.21 and 10.45%. Considering these results in conjunction with consistent evidence from previous literature (18), we recommend implementing community-based public health education campaigns emphasizing the severity of varicella and zoster diseases as well as the protective benefits associated with relevant vaccines. By enhancing public awareness concerning the risks posed by these illnesses alongside the advantages offered by vaccination, it is plausible to foster accurate perceptions about immunization and thereby promote vaccine uptake (33).

Family factors play a significant role in vaccination behaviors. In a survey on awareness of the herpes zoster vaccine, 56.96% of respondents learned about the vaccine through family and friends, highlighting the importance of family and social networks as channels for information dissemination. Furthermore, logistic regression analysis revealed that, compared to individuals with spouses holding a bachelor’s degree or below, those with spouses having a master’s degree or higher were 1.871 times more likely to receive the herpes zoster vaccine. This indicates that the knowledge level of family members can significantly influence vaccination decisions, underscoring the importance of family education (34). However, this finding contrasts with the results of a cross-sectional study conducted in Sweden (35), using decision tree analysis, the study found that among individuals who received vaccination recommendations from healthcare professionals, unmarried individuals exhibited a higher willingness to vaccinate (44.3% vs. 21.2%). This discrepancy may be attributed to the lighter financial burden of unmarried individuals and their greater focus on personal health. In summary, family dynamics and social networks play a crucial role in promoting vaccination behaviors for the herpes zoster vaccine. Families can serve as primary influencers, providing not only information but also emotional encouragement and practical support for vaccination decisions. Initiatives to raise awareness within family networks can amplify the reach of health campaigns and foster a culture of proactive health management.

Vaccination coverage also varies across different demographic characteristics, Simultaneously, the elevated cost of vaccines has significantly diminished the inclination of the populace to undergo vaccination. A study in China revealed that the cumulative incidence and annual incidence rates of herpes zoster are higher in women than in men (6, 36). However, this survey shows that the vaccination coverage among women is lower than that among men. It can be explained by the following reasons. First, women often shoulder responsibilities within families (37), which may leave them with insufficient time to visit vaccination sites. Economic constraints or geographical barriers can also hinder access to vaccination. Second, women may be more susceptible to negative influences from their social circles or families, reducing their trust in vaccines. Wrong information about vaccines may exacerbate their concerns and hesitancy. Third, women’s health decisions may be strongly influenced by male family members or other relatives. If there is resistance to vaccination within the household, this influence may be even more pronounced. This suggests that healthcare personnel should place greater emphasis on women during vaccination campaigns. The herpes zoster vaccine has not yet been incorporated into China’s national immunization program, and its cost (exceeding 1,000 RMB per dose) significantly diminishes residents’ inclination to receive the vaccination. Consequently, it is recommended that the government contemplate integrating the vaccine into the medical insurance system or offering specific subsidies to enhance residents’ willingness to be vaccinated.

This study employed logistic regression and decision tree models to analyze the influencing factors of vaccination behavior for people over 50 years old against herpes zoster. Whereas previous studies have either analyzed the willingness of older adult people to receive the herpes zoster vaccine or the influencing factors of vaccination behavior. This study also has some limitations. The selection of respondents for the sample was conducted from a specific urban or suburban area, which may introduce sampling bias. The data presented in this paper are based on self-reports, and individuals with more frequent interactions with community health service centers may provide more favorable responses. Consequently, the reported vaccination coverage might be overestimated.

In summary, the overall vaccination coverage for the zoster vaccine among residents aged 50 and above is relatively low. It is recommended that government departments should strengthen the integration of medical treatment and prevention, encourage clinicians to prescribe vaccines to suitable populations, and strengthen the key role of medical staff in vaccine promotion. Strengthening community-based public health education activities targeting entire families, including healthy individuals is also suggested. The educational content should concentrate on chickenpox, herpes zoster, and relevant vaccine information, highlighting the risks associated with these diseases and the benefits of vaccination. At last, it is recommended that the government provide subsidies to those who need the herpes zoster vaccine to alleviate the financial burden of vaccination and thereby increase vaccination coverage.

## Data Availability

The raw data supporting the conclusions of this article will be made available by the authors, without undue reservation.

## Footnotes

^1^ http://www.wjx.cn
