# Supplementary material for: A cross-sectional study exploring the predictors of herpes zoster vaccination for people aged over 50 years old in Chaoyang district, Beijing
Source: Front Public Health. 2025 Jan 23;12:1486603. doi: 10.3389/fpubh.2024.1486603 (PMC11799550; doi:10.3389/fpubh.2024.1486603)
Supplement: Supplementary file 1 [file Table_1.docx]

**Supplement materials 1：Questionnaire**

**Survey on the willingness of shingles vaccination in people over 50 years old in Chaoyang District, Beijing**

Hello, we are medical staff of Chaoyang District, Beijing. We are collecting information through this questionnaire to understand the awareness and willingness of shingles vaccination among people over 50 years old in Chaoyang District, and to provide data support for subsequent vaccination. The entire questionnaire is anonymous and takes about 10 minutes. All information in the questionnaire will be confidential. Please feel free to fill in it.

Part 1.

1. Your local community health service center

| ○ urban |
| --- |
| ○ subueban |

2. Your age group

| ○ 50 ~ 59 |
| --- |
| ○ 60 ~ 69 |
| ○ From 70 to 79 |
| ○ From 80 to 89 |

3. Your gender:

| ○ Male | ○ Female |
| --- | --- |

4. Your education:

| ○ Primary School or below | ○ Junior High School | ○ High School/technical secondary school | ○  Undergraduate/Junior College | ○ Master's degree or above |
| --- | --- | --- | --- | --- |

5. Your current occupation or prior to retirement:

| ○ Teacher |
| --- |
| ○ Medical and health workers |
| ○ Personnel of government agencies and public institutions |
| ○ Enterprise personnel |
| ○ Farmers |
| ○ Individual and private personnel |
| ○ Household chores and unemployment |
| ○ Other _________________  Part 2. |

6. Your marital status:

| ○ Single | ○ Married | ○ Divorced |
| --- | --- | --- |
| ○ Widowed and other _________________ |  |  |

7. Your spouse's education:

| ○ Primary school or below | ○ Junior High School | ○ High School/technical secondary school | ○ Undergraduate/Junior College | ○ Master degree or above |
| --- | --- | --- | --- | --- |

Rely on option 2 of question 6

8. How many people are you living with?

| ○1 ~ 2 |
| --- |
| ○3 ~ 5 |
| ○6 or higher |

9. How much is your monthly income (￥):

| ○ 2000 or less | ○ From 2000 to 5000 | ○ From 5000 to 10000 | ○ From 10000 to 15000 | ○ From 15000 to 20000 |
| --- | --- | --- | --- | --- |
| ○ > 20000 |  |  |  |  |

Part 3

10. The status of flu:

| ○ Frequent (twice a year or more) |
| --- |
| ○ Occasionally (once a year) |
| ○ Very rarely (once in many years) |
| ○ Never |

11. Do you suffer from any of the following chronic diseases: hyperlipidemia, hypertension, diabetes, chronic obstructive pulmonary disease, coronary heart disease, chronic kidney disease, systemic lupus erythematosus, chronic eczema, osteoporosis?

| ○ Yes, have been suffering from this disease for several years _________________ |
| --- |
| ○ No |

12. Type of chronic disease you have:

| □ High blood lipids |
| --- |
| □ High blood pressure |
| □ Diabetes |
| □ Chronic obstructive pulmonary disease |
| □ Coronary heart disease |
| □ Chronic kidney disease |
| □ Systemic lupus erythematosus |
| □ Chronic eczema |
| □ Osteoporosis |
| □ Other _________________  Rely on option 1 of question 11  Part 4 |

13. Have you ever heard of the disease shingles (loin wrap/snake wrap)?

| ○ Yes | ○ No |
| --- | --- |

14. Have you ever had chickenpox?

| ○ Yes | ○ No | ○ Unclear |
| --- | --- | --- |

15. Have you ever suffered from shingles (loin wrap/snake wrap)?

| ○ Yes | ○ No | ○ Unclear |
| --- | --- | --- |

16. Has anyone you live with or know very well ever had shingles?

| ○ Yes | ○ No |
| --- | --- |

17. Did you know that people who have had chickenpox can get shingles?

| ○ Yes | ○ No |
| --- | --- |

18. Have you heard of the following herpes zoster vaccine?

| ○ Yes | ○ No |
| --- | --- |

19. How do you know about the herpes zoster vaccine

| □ Newspapers and books |
| --- |
| □ Television |
| □ Internet, mobile phones |
| □ Doctor consultation |
| □ Family and friends |
| □ Social publicity |
| □ Public Lectures |
| □ Never known |

20. Has your health care provider recommended you get the herpes zoster vaccine?

| ○ Yes | ○ No |
| --- | --- |

Part 5

21. Are you willing to be vaccinated against herpes zoster ?

| ○ Yes and have been vaccinated |
| --- |
| ○ Willing but not vaccinated |
| ○ Not willing |

22. Why would you like to get the herpes zoster vaccine?

| □ Strengthen resistance and prevent infectious diseases |
| --- |
| □ Reduce expenditure due to illness |
| □ Vaccination is recommended by the government and health authorities |
| □ Recommended vaccination by relatives and friends |
| □ Medical personnel recommend vaccination |
| □ Others: _________________ |

Relies on # 21 # 1; 2 options

23. The reasons you have not yet received the herpes zoster vaccine are:

| □ Inconvenient vaccination conditions (such as transportation, time, etc.) |
| --- |
| □ The vaccine is too expensive |
| □ Don't know the service information of shingles vaccine |
| □ Objection from family or friends |
| □ Other _________________ |

Rely on option 2 of question 21

24. Why do you not want to get the herpes zoster vaccine?

| □ The vaccine is too expensive |
| --- |
| □ Think you are healthy and don't need vaccinations |
| □ Worried about adverse reactions to vaccines |
| □ They believe that vaccination does little to prevent herpes zoster |
| □ Shingles is considered a minor illness with no serious consequences |
| □ The condition of inoculation is inconvenient |
| □ I have already had shingles and think I don't need to get vaccinated again |
| □ There are contraindications to vaccination |
| □ Lack of information on herpes zoster vaccination |
| □ The vaccine has not been widely used in the country |
| □ Others: _________________ |

Rely on option 3 of question 21

25. The herpes zoster vaccine has been launched in China since 2020, and the price of the vaccine is about 1600 yuan/dose (two doses are required). What is your acceptance of the price?

| Totally unacceptable | ○ 1 | ○ 2 | ○ 3 | ○ 4 | ○ 5 | Very accepting |
| --- | --- | --- | --- | --- | --- | --- |

26 The price range you can accept for the herpes zoster vaccine

| ○ < 100 yuan/dose |
| --- |
| ○100~300 yuan/dose |
| ○300~500 yuan/dose |
| ○500~1000 yuan/dose |
| ○ > 1000 yuan/dose |
